# Elastin molecular aging promotes MDA‐MB‐231 breast cancer cell invasiveness

**DOI:** 10.1002/2211-5463.12455

**Published:** 2018-08-02

**Authors:** Stéphanie Salesse, Ludivine Odoul, Lise Chazée, Christian Garbar, Laurent Duca, Laurent Martiny, Rachid Mahmoudi, Laurent Debelle

**Affiliations:** ^1^ UMR CNRS/URCA 7369 SFR CAP Santé Faculty of Sciences University of Reims Champagne‐Ardenne France; ^2^ Biopathology Department Institut Jean Godinot‐Unicancer Reims France; ^3^ DERM‐I‐C EA7319 Université de Reims Champagne Ardenne France; ^4^ Faculty of Medicine, EA3797 University of Reims Champagne‐Ardenne France; ^5^ Department of Geriatrics and Internal Medicine Maison Blanche Hospital Reims University Hospitals France

**Keywords:** aging, elastin‐derived peptides, invasiveness, matrix metalloprotease‐14, MDA‐MB‐231 breast cancer cells

## Abstract

Elastin is a long‐lived extracellular matrix protein responsible for the structural integrity and function of tissues. Breast cancer elastosis is a complex phenomenon resulting in both the deposition of elastotic masses and the local production of elastin fragments. In invasive human breast cancers, an increase in elastosis is correlated with severity of the disease and age of the patient. Elastin‐derived peptides (EDPs) are a hallmark of aging and are matrikines – matrix fragments having the ability to regulate cell physiology. They are known to promote processes linked to tumor progression, but their effects on breast cancer cells remain unexplored. Our data show that EDPs enhance the invasiveness of MDA‐MB‐231 breast cancer cells through the engagement of matrix metalloproteases 14 and 2. We therefore suggest that elastosis and/or an aged stroma could promote breast cancer cell invasiveness.

**Abbreviations**
DMEM: Dulbecco's modified eagle medium
EDP: elastin‐derived peptide
MMP: matrix metalloprotease
κE: kappa‐elastin

Elastin and collagen are extracellular matrix proteins responsible for the structural integrity and function of tissues. While collagen brings resistance and support to tissues, elastin endows them with their characteristic elasticity properties 1. Both macromolecules are extremely long‐lived. The half‐life of collagen is estimated to be 20 years, that of elastin to be 70 years 2. In contrast to collagen, elastin undergoes virtually no turnover. As a consequence, and because this protein is deposited only during the early stages of life 1, it is assumed that each individual possesses an ‘elastin capital’ that is supposed to last for life 3. Fortunately, elastin is extremely stable and resistant. Nevertheless, during aging, continuous mechanical stress and an increase in elastase activity contribute to the fragmentation of elastic fibers resulting in the release of elastin‐derived peptides (EDPs) 4.

Alteration of elastic fiber biology is one of the hallmarks of aging 5. Aged individuals experience slow and progressive alterations of the elastic functions of their organs, notably in the cardiovascular and respiratory systems. On the grounds of these observations, Robert and colleagues 5 suggested that life expectancy could somehow be limited by elastin aging.

During the past decades, EDPs, which were first envisaged as mere waste products resulting from elastin aging, have been shown to be bioactive compounds regulating numerous biological processes 6. Notably, it has been shown that the presence of EDPs could promote tumor invasion of melanoma 7, fibroblastoma 8, lung carcinoma 9 and glioblastoma 10 cells.

Elastosis is a feature commonly observed in breast cancers 11. Ductal elastosis is associated with carcinoma 12, notably invasive ones 13, 14. Further, it has been reported that elastosis increases progressively with the severity of breast disease 15. Nevertheless, the prognostic value of the occurrence of elastosis is still under discussion as stromal elastosis is correlated with lower Ki67 expression in tumors 11.

Breast cancer elastosis is a complex phenomenon combining both elastin deposition and elastin degradation 16, 17. Thus, elastosis results in both deposition of elastotic masses and local production of elastin fragments. From the matrix point of view, these two components of elastosis need to be distinguished. Indeed, fragments originating from a degraded matrix provide cells with signals that are very different from those given by their parent protein. For instance, stromal cells adhere to elastin 18 but migrate and/or proliferate when they are incubated with elastin fragments 19. This differential behavior is the keystone of the matrikine concept 20, 21.

Therefore, the occurrence of breast cancer elastosis suggests that stromal and tumor cells could be exposed to EDPs, especially in aged subjects as EDP production is a hallmark of aging. Given that these matrikines could change the behavior of several tumor cells 19, we proposed to determine whether EDPs could influence breast cancer cells. The cellular model chosen was the invasive MDA‐MB‐231 cell line. Strikingly, our data suggest that the presence of EDPs contributes to an enhanced invasiveness of these tumor cells by engaging matrix metalloprotease (MMP)‐14. The significance of this finding is discussed.

## Materials and methods

### Reagents

Elastin peptides were prepared as described 22. Briefly, insoluble elastin was prepared from bovine ligamentum nuchae by hot alkali treatment. Comparing its amino acid composition with that predicted from the elastin gene product assessed its purity. Soluble elastin peptides were further obtained from insoluble elastin by organo‐alkaline hydrolysis. This was achieved using 1 m KOH in 80% aqueous ethanol. The obtained mixture of elastin peptides is termed kappa‐elastin (κE) and exhibits the same biological properties as physiological elastin hydrolysates obtained using elastase 23 as it contains several peptides harboring the bioactive GXXPG motif 24. Accordingly, κE is used as a model of EDPs.

Analysis of human and bovine elastin gene sequences demonstrate that they share numerous repetitive sequence similarities 25. Namely, the bovine gene harbors sequence motifs largely found in the human sequence (GXXPG motifs). Thus, while bovine elastin is different from human elastin, peptides obtained from their digestion have comparable structural features 26, 27, making of bovine κE a good model for human elastin peptides.

The circulating concentration of EDPs is estimated to be in the order of ng·mL^−1^ in healthy individuals and μg·mL^−1^ in pathological situations where elastin is fragmented 28, 29, 30. As a consequence, κE is commonly used at 50 μg·mL^−1^ on cells 9.

Rabbit anti‐MMP‐14 antibody was purchased from GeneTex (distributed by Euromedex, Mundolsheim, France). Mouse anti‐MMP‐2 (clone CA‐4001), rabbit anti‐MMP‐14 antibodies, IgGs used as negative controls and FluorSave Reagent were obtained from Merck Millipore (Saint Quentin‐en‐Yvelines, France). Anti‐rabbit AlexaFluor 568 was purchased from Thermo Fisher Scientific (Villebon sur Yvette, France). The metalloproteinase inhibitor 1,10‐phenanthroline monohydrate, the serine proteases inhibitor aprotinin and other chemicals were from Sigma‐Aldrich (Saint Quentin Fallavier, France).

### Cell culture

The human breast cancer cell line MDA‐MB‐231 (ACC732) was purchased from DSMZ (Leibniz Institute DSMZ – German Collection of Microorganisms and Cell Cultures). Cells were cultured in Dulbecco's modified eagle medium (DMEM) supplemented with 10% fetal calf serum (FCS), 100 units·mL^−1^ penicillin and 100 μg·mL^−1^ streptomycin, at 37 °C in a 5% CO_2_ humidified atmosphere. For experiments, MDA‐MB‐231 cells in logarithmic growth phase or at 80% confluence were cultured for 14 h in DMEM without FCS before treatment with 50 μg·mL^−1^ of κE.

### *In vitro* invasion assay

Invasion of breast cancer cells was examined using a modified Boyden chambers technique 31 with 24‐well inserts (8 μm pores) coated with 25 μg of Matrigel^®^ (BD Biosciences, Franklin Lakes, NJ, USA). The lower chambers were filled with 750 μL DMEM supplemented with 10% FCS. MDA‐MB‐231 cells (2 × 10^4^ cells per well) were seeded in the upper compartment of the inserts in 100 μL of DMEM with or without κE (50 μg·mL^−1^) and cultured at 37 °C. The inhibitors aprotinin (100 μg·mL^−1^) and 1,10‐phenanthroline (100 μm), anti‐MMP‐14 (20 μg·mL^−1^) and anti‐MMP‐2 antibodies (20 μg·mL^−1^) were added in the upper chamber with κE. After 6 h of incubation, non‐invasive cells on the upper surface of the filter were wiped off with a cotton swab, while the invasive cells on the lower surface of the filter were fixed with methanol and stained with crystal violet. Invasiveness was determined by counting cells in 10 random fields per well, using light microscopy (Carl Zeiss Axio Observer, Oberkochen, Germany). Each assay was performed in triplicate and repeated at least five times. Due to variation in the number of invasive cells from different experiments, the results were normalized to control conditions.

### Gelatin zymography analysis

MDA‐MB‐231 cells at subconfluence were cultured for 14 h in DMEM without FCS. Cells were then incubated in six‐well plate precoated with Matrigel for 6 h in serum‐free culture medium with or without κE (50 μg·mL^−1^). Conditioned media were harvested and centrifuged at 500 ***g*** for 10 min to remove cellular debris and then concentrated with Vivaspin concentrators (Sartorius, Göttingen, Germany). Conditioned media from HT1080 cell cultures were used as positive controls.

Gelatinase activity in conditioned medium was determined by electrophoresis under non‐reducing conditions on an SDS/polyacrylamide gel containing 0.1% gelatin. After electrophoresis, gels were washed for 1 h at room temperature in a 2.5% (v/v) Triton X‐100 solution to remove SDS, then incubated at 37 °C for 24 h in 50 mm Tris/HCl (pH 7.6) containing 5 mm CaCl_2_, 100 mm NaCl and 0.01% (v/v) Triton X‐100. The gels were then stained with 0.1% (w/v) Coomassie brilliant blue R‐250 (Sigma‐Aldrich) in 45% (v/v) methanol/10% (v/v) acetic acid. After 45 min, the gels were destained with 25% (v/v) methanol/10% acetic acid for 30 min. The proteolytic activity was detected as clear bands on a blue background of the Coomassie brilliant blue‐stained gel.

### RNA isolation and RT‐PCR

Total mRNAs were extracted with TRIzol reagent (Thermo Fisher Scientific) and separated from other cellular materials by using chloroform/isoamyl alcohol (24 : 1) and centrifugation (12 000 ***g***, 4 °C, 15 min). Total mRNAs were then precipitated with isopropanol, washed with 75% ethanol and resuspended in water. The quantity and quality of RNA were determined by spectroscopy and migration on agarose gel. Two hundred and fifty nanograms of total mRNA was reverse‐transcribed using the Verso cDNA kit (Thermo Fisher Scientific) according to the manufacturer's instructions. Real‐time PCR was then performed using an Absolute SYBR Green mix (Thermo Fisher Scientific) and a CFX 96 Touch Real‐Time PCR Detection System (Bio‐Rad, Hercules, CA, USA). The cycle threshold (*C*_t_) values were recorded using Bio‐Rad cfx manager 3.0 software. PCR primers were synthesized by Eurogentec (Liège, Belgium) as follow (5′–3′): for MMP‐2: TCTTCCCCTTCACTTTCCTG and ACTTGCGGTCGTCATCGT; for MMP‐14: AACCAAGTGATGGATGGATAC C and CTCCTTAATGT GCTTGGGGTAG; for RS18: GCAGAATCCACGCCAGTACAA and GCCAGTGGTCTTGGTGTGCT; for RPL 32: CATTGGTTATGGAAGCAACAAA and TTCTTGGA GGAAACATTGTGAG. PCR conditions were 15 min at 95 °C, followed by 40 cycles each consisting of 15 s at 95 °C (denaturation) and 1 min at 60 °C (annealing/extension). PCR efficiency of the primer sets (MMP‐2, MMP‐14, RS18 and RPL32) was controlled via the slope of a standard curve. Results were standardized to RS18 and RPL32 gene expression levels using genex software (Bio‐Rad).

### Immunofluorescence and confocal microscopy

MDA‐MB‐231 cells (1.5 × 10^4^) were seeded onto Matrigel‐coated glass coverslips and cultured for 24 h in DMEM supplemented with 10% FCS at 37 °C. After two washes with PBS, cells were cultured with or without κE (50 μg·mL^−1^) for 6 or 24 h in DMEM without FCS, then fixed in 2% paraformaldehyde, washed with PBS and incubated for 1 h at room temperature in blocking solution (PBS containing 3% bovine serum albumin). Cells were then incubated overnight at 4 °C with MMP‐14 primary antibody (rabbit anti‐human MMP‐14 antibody, clone EP1264Y, GeneTex), washed and stained for 30 min at room temperature with goat anti‐rabbit secondary antibody (Invitrogen/Thermo Fisher Scientific) conjugated to Alexa Fluor 568 and Hoescht reagent. Finally, they were mounted in FluorSave Reagent (Merck, Darmstadt, Germany) and examined using an LSM710 NLO laser scanning confocal microscope (Carl Zeiss Axio Observer). Emitted fluorescence was detected through the use of the appropriate filter set, and representative images from three separate experiments were treated and merged with imagej software (NIH, Bethesda, MD, USA).

### Statistical analysis

The results are expressed as mean ± standard error of the mean. The experiments were performed at least three times. Every individual experiment was performed in triplicate. Statistical significance of differences was assessed using a two‐sided paired Student's *t* test. Results are considered significantly different when the *P*‐value was < 0.05.

## Results

### Elastin‐derived peptides promote MDA‐MB‐231 cell invasion

Elastin‐derived peptides exhibit numerous biological activities linked to cancer progression 6. For instance, it has been shown that their presence could promote tumor invasion of melanoma 7, fibroblastoma 8, lung carcinoma 9 and gliobastoma 10 cells. Strikingly, their influence on breast cancer cells remained undocumented.

As a consequence, the first objective of the present study was to determine if EDPs could have an effect on breast cancer cells. To this end, we analyzed their effect on the MDA‐MB‐231 cell line, which is a cellular model for triple‐negative tumors 32. We evaluated the consequence of their presence for cell adhesion, proliferation, migration and invasiveness.

**Figure 1:**
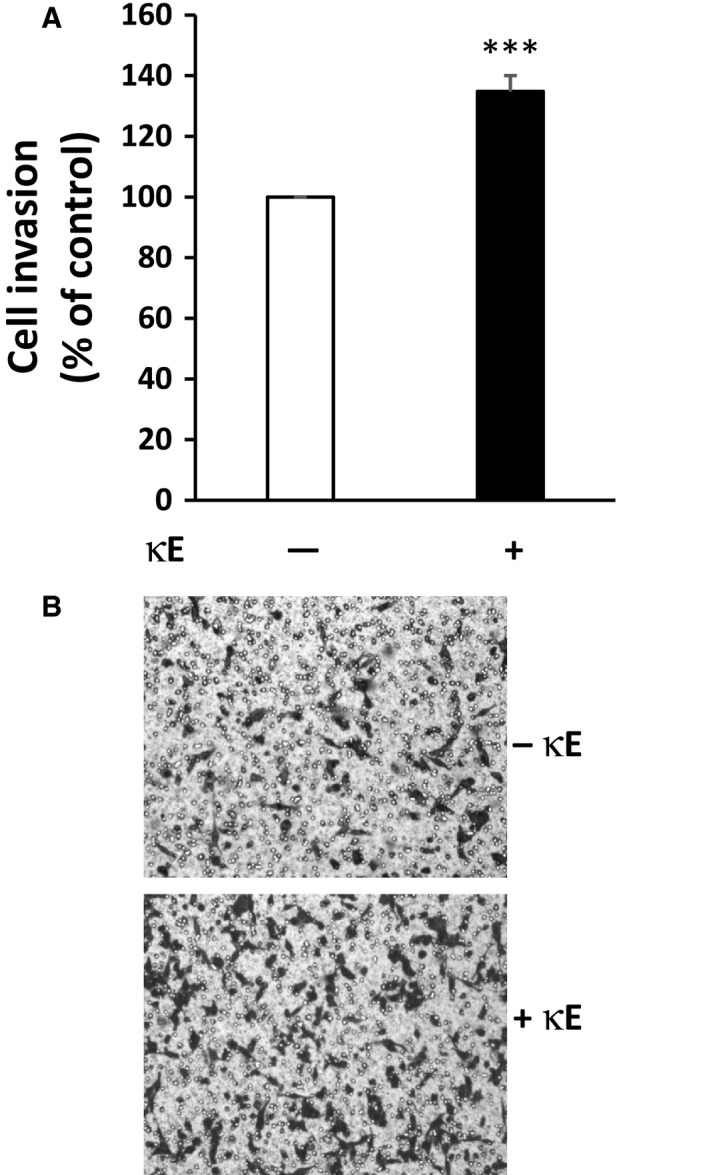
Elastin‐derived peptides promote MDA‐MB‐231 cancer cell invasiveness. Cells were incubated for 6 h in the presence or absence of κE (50 μg·mL^−1^) in a modified Boyden chamber in order to assess their invasiveness. Cells were placed in the upper compartment of the system in the presence of the agonist while the lower compartment received only culture medium supplemented with 10% serum. Cells having invaded the Matrigel separating the two compartments and being present on its lower surface were counted after 6 h. (A) Invasiveness expressed as the percentage of the control, here MDA‐MB‐231 cells in the absence of κE. (B) Representative images of violet crystal‐stained invasive cells. ***P < 0.001 (*n* = 13).

We observed that EDPs had no significant effect on MDA‐MB‐231 cell adhesion (Fig. S1). Likewise, EDPs failed to promote cell proliferation at 24 h of culture but a significant difference was observed at 48 h and beyond (Fig. S2). As a consequence, we later made our invasion measurement at 6 h in order to avoid any unwanted effects due to cell proliferation. Finally, our data indicated that EDPs treatment had no effect on MDA‐MB‐231 cell migration (Fig. S3). In contrast, we observed that EDP treatment had an impact on MDA‐MB‐231 cell invasion (Fig. 1). In these experiments, the addition of EDPs (κE, 50 μg·mL^−1^) in the upper compartment of the transwells where the tumor cells were present resulted in a significant increase (about 34%) in their capacity to invade the Matrigel.

This result therefore suggested that EDPs could promote invasiveness of MDA‐MB‐231 breast cancer cells and thus enhance their aggressiveness.

### EDP‐induced MDA‐MB‐231 cell invasion relies on MMP involvement

In order to have further insights into the cellular events explaining the increased invasiveness of MDA‐MB‐231 cells in the presence of EDPs, we analyzed the contribution of protease cascades to this phenomenon.

The MMPs and Ser‐proteases are critically involved in tumor invasion 33. In order to check whether these protease cascades are involved in the invasion process promoted by EDPs, we stimulated MDA‐MB‐231 cells in the presence of pan‐inhibitors of these protease activities, namely aprotinin (100 μg·mL^−1^) for Ser‐proteases and 1,10‐phenanthroline (100 μm) for MMPs.

**Figure 2:**
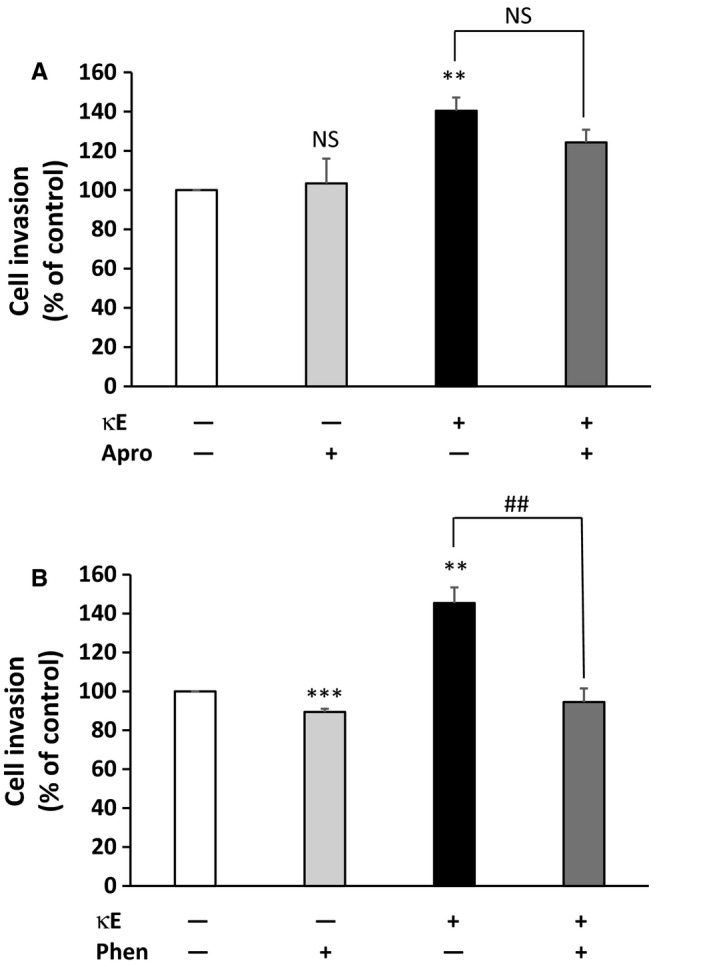
Effect of proteases inhibition on EDP‐induced invasiveness of MDA‐MB‐231 cells. Cells were incubated for 6 h in the upper compartment of the modified Boyden chamber in the presence or absence of κE (50 μg·mL^−1^) and inhibitors. Cells having invaded were counted after 6 h. Invasiveness is expressed as a percentage of the control, here MDA‐MB‐231 cells in the absence of κE. (A) Inhibition of Ser‐proteases with 100 μg·mL^−1^ aprotinin (Apro). (B) Inhibition of MMPs with 100 μm 1,10‐phenanthroline (Phen). Labels above the bars indicate the following: *comparison with the control (white bar); ^#^comparison between the indicated experimental conditions. **,^##^ *P* < 0.01; ***P < 0.001; NS, not significant (*n* = 3).

As shown in Fig. 2A, the inhibition of the Ser‐proteases did not significantly block EDP‐induced MDA‐MB‐231 cell invasion. This observation suggested that Ser‐proteases were not involved in EDP‐induced MDA‐MB‐231 cell invasion.

We thus analyzed the involvement of MMPs (Fig. 2B). When cells were treated with 1,10‐phenanthroline alone, the capacity of MDA‐MB‐231 cells to invade was slightly reduced. This significant decrease was expected as MDA‐MB‐231 cells are invasive and therefore express several MMPs. Strikingly, when cells were stimulated by EDPs in the presence of 1,10‐phenanthroline, the invasive capacity of the cells was significantly reduced and returned to a level comparable to the control. These data strongly suggested that the invasive capacity exhibited by MDA‐MB‐231 cells in the presence of EDPs relied on MMP catalytic activity. We therefore concluded that the engagement of the MMP cascade can explain the increased invasiveness of MDA‐MB‐231 cells in the presence of EDPs.

### EDP treatment increases MMP‐14 cell surface levels in MDA‐MB‐231 cells

Among MMPs, MMP‐2 (gelatinase A) and MMP‐14 (MT1‐MMP) have been linked to cancer invasion promoted by EDPs 9, 23, 34. While MMP‐2 can be released by cells to degrade their surrounding matrix, MMP‐14 is strictly a membrane‐associated protease 35.

MMP‐14 is of particular interest because this enzyme is involved in multiple events linked to breast cancer invasion, such as basement membrane breaching, tumor invasion, intracellular trafficking and cell motility regulation 35.

**Figure 3:**
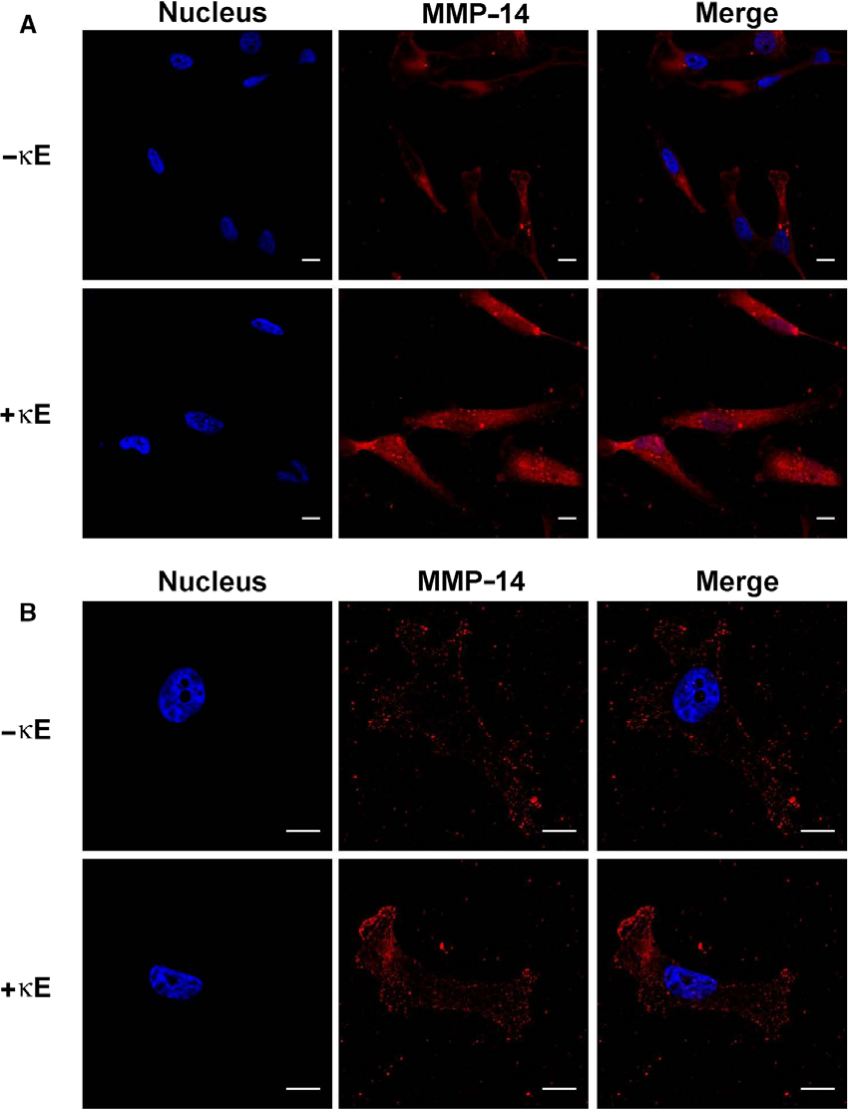
Confocal imaging of MDA‐MB‐231 cells reveals an increased level of MMP‐14 at the surface of EDP‐treated cells. Cells were cultured on Matrigel in the presence or absence of κE (50 μg·mL^−1^) for 24 h (A) or 6 h (B). MMP‐14 was imaged by confocal microscopy using an AlexaFluor568‐coupled anti‐MMP‐14 antibody. MMP‐14 labelling appears red and cell nuclei appear blue following Hoechst staining. All images are representative of three independent experiments. Scale bars, 10 μm.

In the absence of EDPs, MDA‐MB‐231 cells evidenced a faint MMP‐14 labelling consistent with their invasive phenotype. The level of MMP‐14 present on the cell surface was considerably higher when these cells were cultured in the presence of EDPs for 24 h (Fig. 3A). Strikingly, this increase in MMP‐14 level at the cell surface was also observed when MDA‐MB‐231 cells were treated with EDPs for 6 h (Fig. 3B). Altogether these observations suggest that EDP treatment increases the presence of MMP‐14 at the cell surface of the breast cancer cells. This assumption was in good agreement with the data obtained previously, but at that point we could not link MMP‐14 directly to the observed increased invasiveness of MDA‐MB‐231 cells in the presence of EDPs.

### EDP treatment increases MMP‐2 secretion and activation by MDA‐MB‐231 cells

**Figure 4:**
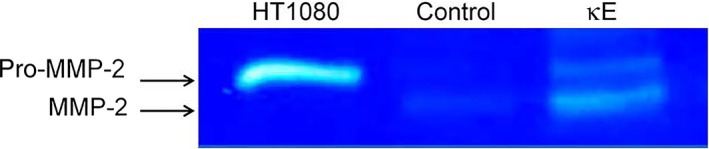
Elastin‐derived peptides induced MMP‐2 secretion by MDA‐MB‐231 cells. Gelatinolytic activity (72 kDa, pro‐MMP‐2; 62 kDa, MMP‐2) was evaluated by gelatin zymography in 6 h‐conditioned media of MDA‐MB‐231 cells treated (κE) or not treated (Control) with κE (50 μg·mL^−1^). HT1080 cell line conditioned medium was used as positive control 36. The zymogram image is representative of three independent experiments.

MMP‐2 secretion after EDP treatment of MDA‐MB‐231 cells for 6 h was investigated by zymography. As shown in Fig. 4, these cells secreted MMP‐2 after κE treatment but not in the absence of treatment. Strikingly, MMP‐2 existed in both the inactive pro‐MMP‐2 and active MMP‐2 forms when MDA‐MB‐231 cells were exposed to EDPs. As MMP‐2 was mostly in its active form, this finding suggested that MMP‐2 could be involved in the enhanced EDP‐induced MDA‐MB‐231 invasive behavior.

### Effect of EDPs on MMP‐2 and MMP‐14 expression in MDA‐MB‐231 cells

**Figure 5:**
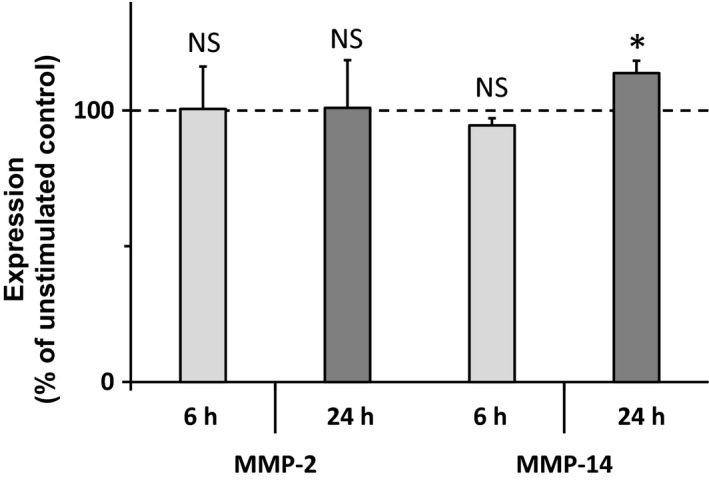
Effect of EDPs on MMP‐2 and MMP‐14 gene expression. RT‐qPCR analysis of MMP‐2 and MMP‐14 mRNA levels was performed from MDA‐MB‐231 cells cultured 6 h (*n* = 5) and 24 h (*n* = 3) in the presence or absence (Control) of κE (50 μg·mL^−1^) and normalized to the RS18 and RPL32 mRNA values. Data are expressed as percentages of the corresponding unstimulated control. NS, not significant; *P < 0.05.

As EDPs had effects on both MMP‐14 and MMP‐2 protein levels, we further determined whether EDPs could also up‐regulate their expression (Fig. 5). After 6 h of treatment, neither MMP‐2 nor MMP‐14 RNA level was modified as compared with their levels in the absence of EDPs. In contrast, a slight, but significant, increase of MMP‐14 RNA level was observed at 24 h suggesting that EDPs could promote MMP‐14 gene expression at this time point.

### MMP‐2 and MMP‐14 are involved in EDP‐induced MDA‐MB‐231 cell invasion

**Figure 6:**
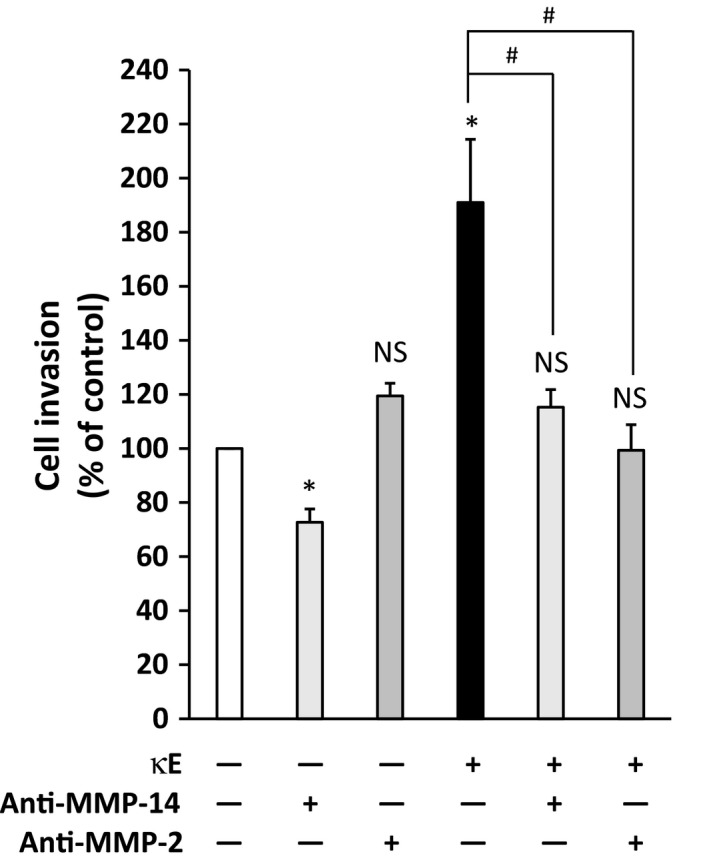
Sequestration of MMP‐14 and MMP‐2 activities by selective antibodies blocks EDP‐induced MDA‐MB‐231 cell invasiveness. Cells were placed in the upper compartment of the Transwell system in the presence or absence of the corresponding anti‐MMP antibody (rabbit anti‐MMP‐14 or mouse anti‐MMP‐2, 20 μg·mL^−1^ each). The agonist (κE) was added (or not) simultaneously in the upper chamber, and cells are incubated for 6 h. Cells having invaded were counted after that period. Invasiveness is expressed as the percentage of the control, here MDA‐MB‐231 cells in the absence of κE. Labels above the bars indicate the following: *comparison with the control (white bar); ^#^comparison between the indicated experimental conditions. *,^#^ *P* < 0.05; NS, not significant (*n* = 3).

In order to assess the role of MMP‐14 and MMP‐2 in EDP‐induced MDA‐MB‐231 cell invasiveness, we used antibodies directed against these enzymes to selectively block their contribution to this effect (Fig. 6). The data indicate that the use of the anti‐MMP‐14 alone slightly, but significantly, reduced MDA‐MB‐231 cell invasiveness. This result is consistent with the invasive phenotype and the fact that these cells express MMP‐14 at their surface. When cells were treated in the presence of both anti‐MMP‐14 and EDPs, we observed that the EDP‐promoted invasiveness was abolished as no significant effect of EDPs was evident.

Similar results were obtained blocking MMP‐2 activity except that the anti‐MMP‐2 antibody alone had no effect. In both cases, incubating the cells with an IgG having the corresponding antibody isotype alone had no effect on cell invasiveness (data not shown).

Altogether, these results clearly indicated that MMP‐14 and MMP‐2 activities are involved in EDP‐induced MDA‐MB‐231 cell invasiveness.

## Discussion

The extracellular matrix has long been considered a support tissue for cells. It is now well established that this intricate network of fibers is extremely dynamic and matrix remodeling is a common term used to describe the interplay of synthesis and degradation characterizing matrix biology 1. As long as this equilibrium is balanced, matrix homeostasis is achieved and the supported tissue behaves as it should. However, should this balance be disrupted, a pathological situation can occur. Late stages of chronic obstructive pulmonary disease and formation of aneurysms exemplify this situation.

Actually, matrix degradation is rarely massive. More often it is a localized phenomenon with a limited duration. Wound healing is a good illustration of this controlled matrix remodeling. Nevertheless, matrix degradation can also be a chronic event. This is typical of elastin biology. Elastin is deposited in the early stages of life, providing an elastin capital for life. Although elastin is an extremely resistant molecule, its permanent subjection to mechanical strain together with aggression by elastases lead it to age and break. Elastin aging is thus translated into loss of elasticity, which is a visible marker of aging, but is also seen in the constant release of elastin‐derived peptides 3, 4.

It has now been demonstrated that EDPs are not mere waste products. These peptides possess intrinsic biological activity some of which is directly linked to tumor progression 6. As a consequence, the aim of this work was to explore the possibility that EDPs could contribute to breast cancer invasiveness in the context of an aging tissue. In this study, tissue aging was simulated by the addition of EDPs to cells.

Our data indicate that EDPs have no effect on MDA‐MB‐231 cell proliferation, adhesion on gelatin and migration (Figs [Link], [Link], [Link]). In contrast, their ability to invade a matrix environment was enhanced in the presence of EDPs (Fig. 1). Our results demonstrate that Ser‐proteases are not involved in this process while the MMP cascade is (Fig. 2).

Interestingly, EDPs increased the levels of MMP‐14 at the cell surface of MDA‐MB‐231 cells (Fig. 3), while engagement of MMP‐14 following EDP treatment had only been evidenced for fibrosarcoma 36 and endothelial cells 37.

As already reported in fibrosarcoma 36, melanoma 38 and lung carcinoma 9, we observed that EDPs promoted MMP‐2 secretion and activation in MDA‐MB‐231 cells (Fig. 4). Strikingly, the expression level of MMP‐2 was not altered by EDP treatment while a slight increase of MMP‐14 expression could be observed at 24 h (Fig. 5). As a consequence, we conclude that EDPs could upregulate MMP‐14 expression and availability in these cells. Finally, we proved that MMP‐14 and MMP‐2 are both involved in the increased invasiveness observed after EDP treatment as the selective blockade of these proteases blocked the effects of EDPs on invasiveness (Fig. 6). Strikingly, the selective inhibition of MMP‐2 or MMP‐14 returned the cell invasion level to that of the control suggesting that MMP‐2 and MMP‐14 activations could be linked.

The activation of MMP‐2 by MMP‐14 has been reported in the presence of TIMP‐2 39. MDA‐MB‐231 cells express TIMP‐2 (data not shown). In our conditions, this activation scheme of MMP‐2 by MMP‐14 is likely. The accumulation of active forms of MMP‐2 in the conditioned medium of treated cells (Fig. 4) supports this view. Nevertheless, our work does not constitute a clear demonstration. The link between MMP‐14 and MMP‐2 activation has therefore to be explored further.

Taken together, our data suggest that EDPs could promote invasion of triple‐negative cancer cells via MMP‐14 and MMP‐2. As a consequence, this work suggests that an environmental factor derived from an aged stroma or elastosis (elastin fragments) could possibly influence breast tumor progression by mobilizing greater amounts of active MMPs thereby modifying tumor cell invasive capacity.

## Conclusion

Our data provide new information on the place of EDPs in breast tumor progression. Indeed, these matrikines are characteristic of an aging environment and, in breast cancer, may also derive from the matrix remodeling occurring during elastosis.

Elastin‐derived peptides possess numerous biological activities towards both cancer cells and their surrounding stroma. They potentiate the migration and matrix invasion of tumor cells 8, 9, 10, 36, 37. They stimulate the migration and proliferation of monocytes and skin fibroblasts 40, 41. They up‐regulate MMP expression by fibroblasts inducing a remodeling program in favor of melanoma cell invasion 7. Additionally, they are pro‐angiogenic 37, 42, 43, chemotactic for inflammatory cells 44, 45, 46, 47 and promote elastase release 48, 49, 50. Finally, EDPs are strong survival signals as they promote resistance to apoptosis 51.

As shown in this work, EDPs promote MDA‐MB‐231 breast cancer cell invasiveness. Thus, our data suggest that EDPs could be important players in the breast tumor environment. For all these reasons, we feel that the effect of these matrikines on mammary tumors and their surroundings can no longer be overlooked, especially when aged subjects are considered.

## Author contributions

SS, LDe and LDu conceived the study; SS and LDe supervised the study; LO and SS performed the experiments; LC performed MMP‐2 zymography experiments; SS, LO and LDe analyzed the results; LDe and SS wrote the manuscript; LM, CG and LDu revised the final manuscript.

## Supporting information


**Fig. S1.** Influence of EDPs on MDA‐MB‐231 cancer cell adhesion.

**Fig. S2.** Influence of EDPs on MDA‐MB‐231 cell proliferation.

**Fig. S3.** Effect of EDPs on MDA‐MB‐231 cell migration.
