# Supplementary material for: Elastin molecular aging promotes MDA‐MB‐231 breast cancer cell invasiveness
Source: FEBS Open Bio. 2018 Aug 2;8(9):1395–404. doi: 10.1002/2211-5463.12455 (PMC6120250; doi:10.1002/2211-5463.12455)
Supplement: Supplementary file 1 — Fig. S1. Influence of EDPs on MDA‐MB‐231 cancer cell adhesion. [file FEB4-8-1395-s001.pdf]

Figure S1. Influence of EDPs on MDA-MB-231 cancer cells adhesion.

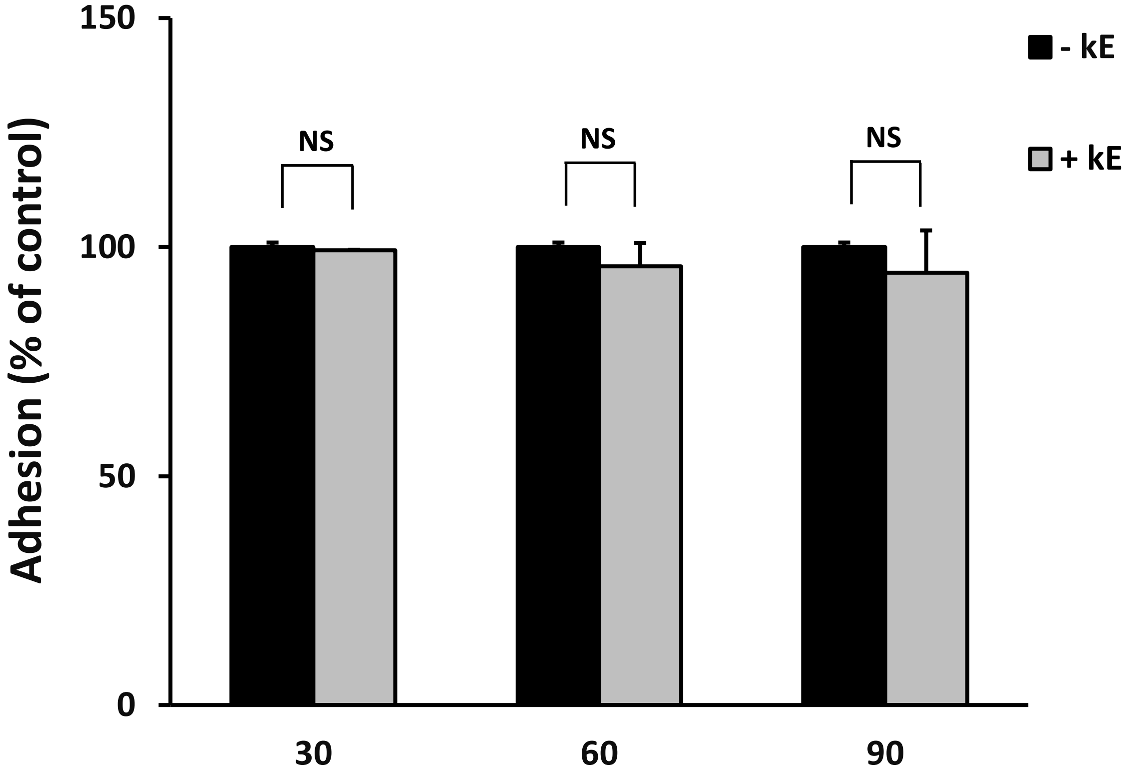

The capacity of MDA-MB-231 cells to adhere onto gelatin in the presence or absence of kE (50 µg/ml) has been evaluated for 30, 60 and 90 min. Adhering cells were then fixed and colored using Violet Crystal. Adhesion of treated cells is expressed as a percentage of the corresponding control without kE. The presented histogram was established from 2 independent series of experiments (n=2). NS, not significant.

As observed, the treatment of cells with kE does not change their ability to adhere onto gelatin. We therefore concluded that EDPs had no effect on MDA-MB-231 cells adhesion.
