# Supplementary material for: Elastin molecular aging promotes MDA‐MB‐231 breast cancer cell invasiveness
Source: FEBS Open Bio. 2018 Aug 2;8(9):1395–404. doi: 10.1002/2211-5463.12455 (PMC6120250; doi:10.1002/2211-5463.12455)
Supplement: Supplementary file 2 — Fig. S2. Influence of EDPs on MDA‐MB‐231 cell proliferation. [file FEB4-8-1395-s002.pdf]

Figure S2. Influence of EDPs on MDA-MB-231 proliferation.

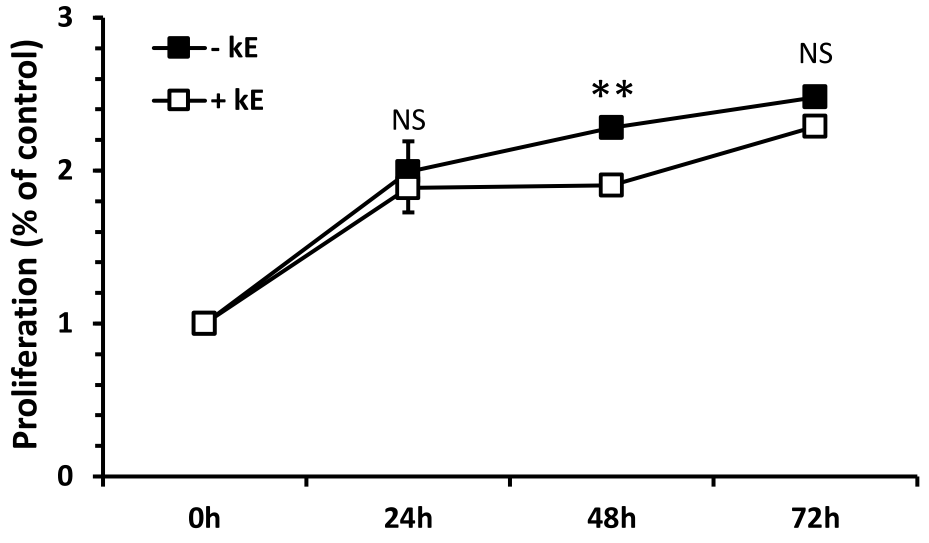

Cells were treated with kE (50 µg/ml) for 24h, 48h and 72h. The effect of EDP was assessed after this period of incubation using a MTT test (ThermoFisher Scientific, Montigny-le-Bretonneux, France). The proliferative capacity of MDA-MB-231 cells is expressed as a percentage of the control without kE at the beginning of the incubation. Three independent experiments were performed (n=3). NS, not significant; **, $p<0.01$.

Our results show that the proliferation of MDA-MB-231 cells is changed when cells are cultured in the presence of kE for 24h. The difference is small but significant out of three independent experiments. At 6h, no significant variation could be observed between MDA-MB-231 cells cultured in the presence of kE and those grown without. Therefore, in order to exclude any possible bias derived from altered MDA-MB-231 proliferation, the effect of kE on these cells was evaluated after 6h of incubation in all subsequent experiments.
