# Supplementary material for: Elastin molecular aging promotes MDA‐MB‐231 breast cancer cell invasiveness
Source: FEBS Open Bio. 2018 Aug 2;8(9):1395–404. doi: 10.1002/2211-5463.12455 (PMC6120250; doi:10.1002/2211-5463.12455)
Supplement: Supplementary file 3 — Fig. S3. Effect of EDPs on MDA‐MB‐231 cell migration. [file FEB4-8-1395-s003.pdf]

Figure S3. Effect of EDPs on MDA-MB-231 cells migration

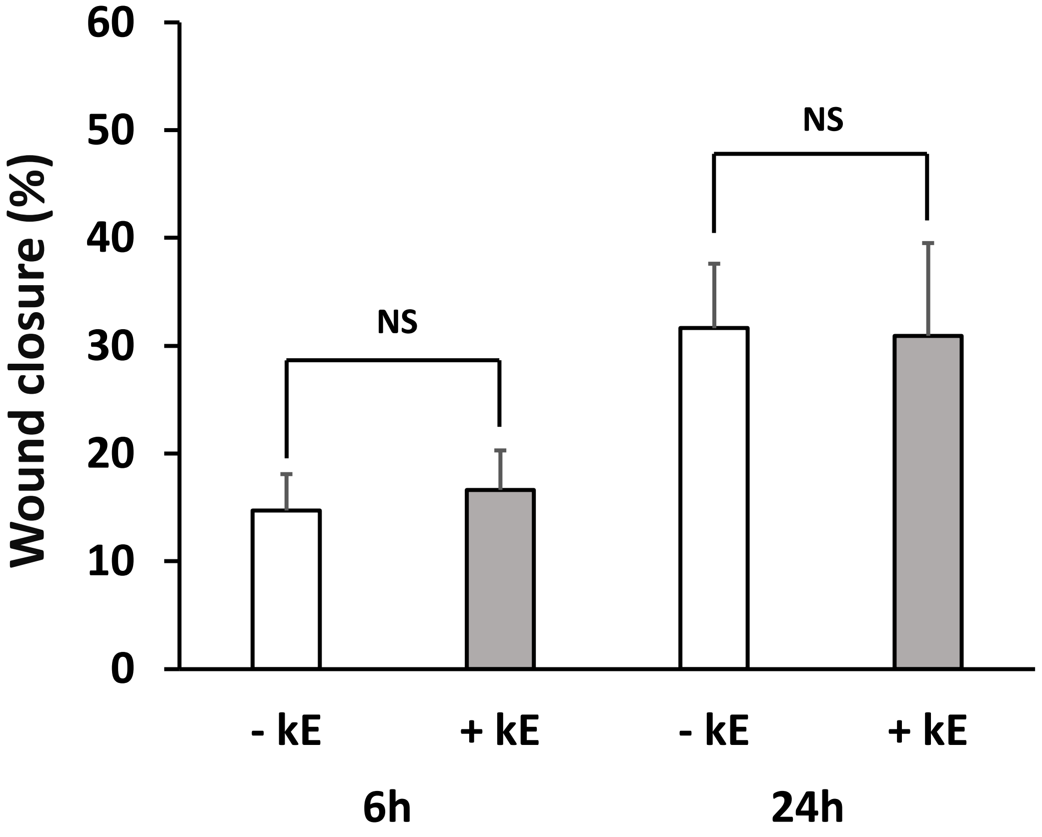

Cell migration was evaluated using a classic scratch wound assay. A wound was made in MDA-MB-231 cell layer and migration was allowed in a medium containing either nothing or kE (50 µg/ml). The area of the wound was measured at the corresponding times using the wound healing tool implemented in the ImageJ software. The results are expressed as a percentage of wound closure (n=3). NS, not significant.

As observed, the presence of EDPs had no impact on the capacity of MDA-MB-231 to close the wound. We therefore concluded that EDPs did not influence MDA-MB-231 cells migration.
